# Supplementary material for: Assessing math anxiety in elementary schoolchildren through a Spanish version of the Scale for Early Mathematics Anxiety (SEMA)
Source: PLoS One. 2021 Aug 5;16(8):e0255777. doi: 10.1371/journal.pone.0255777 (PMC8341591; doi:10.1371/journal.pone.0255777)
Supplement: S1 File — (DOCX) [file pone.0255777.s002.docx]

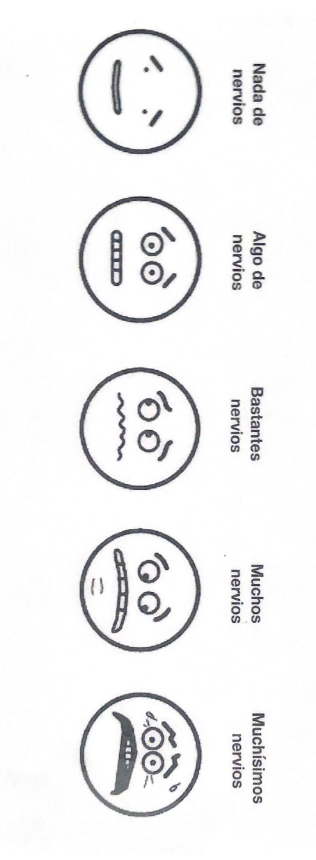


S1 File. Graded anxious faces and their correspondent written options (from left to right) from the SEMA: (i) “Nada de nervios” (not nervous at all); (ii) “Algo de nervios” (a little nervous); (iii) “Bastantes nervios” (somewhat nervous); (iv) “Muchos nervios” (very nervous); and (v) “Muchísimos nervios” (very very nervous). Images reproduced with permission of the authors [1].

References

1. Wu S, Amin H, Barth M, Malcarne V, Menon V. Math anxiety in second and third graders and its relation to mathematics achievement. Frontiers in psychology. 2012;3: 162.
