# Supplementary material for: Assessing math anxiety in elementary schoolchildren through a Spanish version of the Scale for Early Mathematics Anxiety (SEMA)
Source: PLoS One. 2021 Aug 5;16(8):e0255777. doi: 10.1371/journal.pone.0255777 (PMC8341591; doi:10.1371/journal.pone.0255777)
Supplement: Attachment — Submitted filename: Response to Reviewers.docx [file pone.0255777.s003.docx]

April 15^th^, 2021

Dear editor,

We are very grateful for having the opportunity to revise our manuscript entitled “Assessing math anxiety in elementary schoolchildren through a Spanish version of the Scale for Early Mathematics Anxiety (SEMA)”. In the revised manuscript, we have carefully considered the reviewers’ comments and suggestions and have also taken the chance to improve the quality of the paper. As instructed, we have replied to each comment raised by the three reviewers (the reviewer’s comments have been kept in black ink whereas our answers are in blue color), and uploaded also the revised manuscript in two versions: one marked-up copy highlighting the changes made to the original version and another unmarked version of the revised paper without tracked changes.

The reviewers’ comments were very helpful, and we thank them for their constructive and generous feedback on our submission. After addressing all reviewers’ concerns, we feel the quality of the paper is much improved and suitable for publication in Plos One journal.

Best regards,

Dr. Noelia Sánchez-Pérez

First of all, we have to say that after we received the Editor’s decision letter some missed data from children that participated in the study were found. This has led us to have to re-analyze all data including those collected from the new children. For that reason, the sample size has changed slightly. Fortunately, the results, although with minor changes in some decimal places, remain overall as in the original version we submitted to the journal. Specifically, the results that have changed referred to:

- ANCOVAs analyses with trait anxiety as a covariate (lines 312-329, page 14; the lines and pages correspond to the unmarked version of the revised paper without tracked changes.). In the previous version, there were marginal differences in global MA, numerical processing, and the situational and performance anxiety factors. With the changes in the sample those marginal effects were no significant.
- The hierarchical regressions to predict math test scores (calculation, and math fluency) with numerical processing anxiety factor as a IV (lines 439-448, table 6, page 20). In the previous version, numerical processing anxiety factor was a significant predictor of calculation and math fluency. Although it remains as a significant predictor for math fluency, its contribution to calculation is not significant in the new version of the manuscript.

**Reviewer #1**

 I appreciate that the author did a rigorous and detailed statistical analysis (e.g., ANCOVA, CFA) to address the research questions. However, I have a major concern regarding the novelty of the study. To be honest, I don't see much novelty for this study merely looking at math anxiety, trait anxiety, math performance, and non-verbal IQ. Simply translating SEMA to Spanish contributes little to the extant research.

We thank the reviewer for taking the time to review the paper. We are sorry for not being able to attract his/her interest. Our main purpose was to develop an instrument to identify math anxiety among elementary school students in Spain, and to study its psychometric properties. In undertaking this task, the following issues concerning the study of MA in children have been addressed: the measurement of MA in children; the structure of MA at this age period; age and gender differences; the association of MA with anxiety trait; and MA relations with mathematics performance. However, more than a simple translation of the SEMA into Spanish, we aimed to explore the usefulness of this instrument to predict math performance from a broader perspective that includes both standardized math tests and teachers’ scores, as well as in a range of ages beyond those previously explored. As it will be described later on in our reply to the other reviewers’ concerns, from our point of view our results make an important contribution in this respect.

The new version of the manuscript addresses more seriously the adequacy of the SEMA to measure MA in upper elementary school students by comparing the reliability as well as the structural validity of the instrument in older children compared to younger ones, as well as identifying the contribution of the SEMA scales in the prediction of math performance in older compared to younger students. Another issue more profoundly studied concerns the factorial structure of MA as measured through the SEMA; we have paid deeper attention to the fact that the numerical processing anxiety and situational and performance anxiety factors are highly correlated, and have tested the adequacy of a unifactorial model versus a two-factor model by comparing the respective fit indices provided for the whole sample and splitting the sample by boys/girls, and younger/older children. Additionally, the appropriateness of using a broad measure of MA versus more specific ones is considered in connection with math performance. Finally, and following the other reviewers’ suggestions, we have introduced a great deal of changes in the different sections of the paper that hopefully have improved the quality and the interest of the manuscript.

Also, I am pretty concerned about some conclusions the author drew. For example, under "Math anxiety and math performance", the author stated "the Situational and Performance factor negatively predicted students’ grades 423
given by teachers..." **I don't think this could actually "predict**", given the lack of evidence of causality.

To make the predictivity issue clearer to the readers we have introduced the specific time/month when the data were collected in the procedure (line 262-267, page 11; the lines and pages correspond to the unmarked version of the revised paper without tracked changes). Specifically, math anxiety scales were collected between October and December 2018, achievement standardized tests between January and Mach 2019, whereas children’s math grades were informed in June 2019. Although we acknowledge causality is a common issue in this sort of studies, we think we are allowed to use the term “predict” in a temporal sense because math anxiety was measured at the beginning of the academic year whereas the math performance measures were obtained later.

**Reviewer #2**

I enjoyed reading this manuscript. Overall, the study provides useful insight into maths anxiety in a young Spanish population. I particularly like the range of maths attainment measures, especially (as the authors point out), studies tend not to include such a wide range. The usefulness of this is emphasized by the regression findings where different patterns of results are shown. The manuscript reads well and I believe the work will add sufficiently to the literature in this field. However, there are **several grammatical/wording errors** throughout and a thorough proof read to ensure correct English is required before publication. I have made a few minor comments on individual sections below.

We thank the reviewer for his/her kind comments about the manuscript. We apologize about the grammatical/wording errors. They have been corrected in the new version of the paper.

Intro
“**decreasing levels of experienced anxiety 82 when facing mathematics operations throughout childhood would be expected due to the development of executive functioning**”. I am not sure of the logic here given that difficulty level is increased throughout schooling, in line with such development.

We agree that our expectation about how MA would develop throughout school years was rather naïve, bearing in mind that MA can be affected by a diversity of endogenous and exogenous factors, and not only developmental ones. On another side, as it was pointed out by the third reviewer, changes in the scores across grades of global MA as well as the Numerical Processing factor are difficult to interpret because of the different difficulty that specific arithmetic problems involve for students of so different grade levels. For this reason, although we have kept the ANOVAS involving grade to inform about how the scores of the SEMA scales change with grade, we have decided not to fathom deeply on the interpretation of such changes. Nonetheless, we have introduced this issue referring to the work by Sorvo et al., (2017), who attributed the decreasing mean levels of MA as grade increased to the students’ familiarization with the study of math (see lines 93-95, page 4; the lines and pages correspond to the unmarked version of the revised paper without tracked changes).

Participants
Rather than specifying exact salary, it would help to put the salary breakdown into context, e.g. referring to percentages below, at, or above the **mean/median national salary**.

In order to put the income level into context, we have introduced the mean monthly family incomes of Region of Murcia, the geographical area in which the study is placed (lines 214-215, page 9). It was not possible to express the information as requested by the reviewer because the questionnaire administered did not ask the families to exactly inform about their monthly incomes but rather asked them to choose one among 6 options that expressed different incomes intervals.

Results
The T-tests on gender are not needed alongside reporting the main effect of gender from the ANOVAs.

We agree with the reviewer, so we have eliminated the *t*-test analyses, but kept the information about the mean and standard deviation for boys and girls.

pp 295-297 – it would help to be more explicit that “total score” refers to total maths anxiety score.

We agree that the term “total score” is very unspecific. Instead, we now use the term “global MA” to refer to the total math anxiety score.

Discussion
In the subsection “maths anxiety and its relation to general anxiety”, to further support the argument for differentiating trait anxiety from maths anxiety, **it might be worth highlighting how trait anxiety was no longer a significant predictor in the regression models (on performance) when maths anxiety was included.**

We thank the reviewer for highlighting this important issue. We have introduced this fact to stress that these two constructs, although correlated, are separate entities (lines 556-558, page 26).

**Reviewer #3**

My main concern is the **use of a math anxiety measure that has math problems in the questions when using the measure across development**. This makes it very difficult to understand the reason behind grade level differences. In addition, I think more information is needed about the items on the scale and the **reasoning for removing any items**.

We are aware that the items of the Numerical Processing anxiety scale contain math problems and operations that are learned in early elementary school and that these problems involve different levels of difficulty to younger compared to older children. However, our position is that this scale, although not suitable to compare the mean levels of MA across grades, can be useful to detect MA in children in the upper elementary years. We based this assumption on research carried out in adult population in which the stimuli (and items in questionnaires) used to uncover math anxious individuals are simple arithmetic operations, and cognitively undemanding tasks (see lines 179-183, page 8; the lines and pages correspond to the unmarked version of the revised paper without tracked changes).

We have decided to keep the ANOVAS involving grade because we believe that showing how the scores of the different SEMA scales vary with grade are informative of the functioning of the SEMA in the sample studied. However, we have warned the reader about the limitation of Numerical Processing to identify true differences in MA across grades. (see lines 523-526, page 25).

We have also provided more information about the reason why we removed items 2, 4, 6, 12, and 13, as addressed below.

Introduction
- line 111: Can you talk more about **what issues need to be thought about to make sure that a scale is developmentally appropriate for children**?

Following the excellent review by Ganley and McGraw (2016), we have added a new paragraph describing the aspects that should be taken into consideration when generating instruments directed to children. We thank the reviewer for this suggestion because in the new version of the manuscript readers could better appreciate the efforts involved in the development of such measurement instruments (lines 123-139, page 6).

Method
- line 142: Can you mention what percentage of families answered the socioeconomic and education-related questions?

Following the suggestion of the reviewer, we have introduced the percentage of families that answered the socioeconomic and education-related questions (lines 199-211, page 9).

- line 158: Including math problems in math anxiety questions when doing research either longitudinally or cross-sectionally across ages can cause problems due to the relative ease or difficulty of the math problems for children of different ages. You end up bringing this up later on, but to me this is the largest issue with this measure (an issue with the original SEMA as well, not just your version). This is especially a problem because one entire sub scale has math problems, and the other one doesn’t. So differences in findings between the subscales are difficult to interpret as well.

We know that the SEMA was designed for early elementary school students, but we wanted to test its usefulness in upper elementary school too. For that reason, besides the general analyses run with the whole sample, we have run additional analyses in which the sample was divided into younger (3^er^, 4^th^ grade), versus older (5^th^, 6^th^ grade) children. For instance, the factorial structure of the SEMA was tested for younger and older children. We also considered the relative predictive power of MA on math performance depending on the school stage. On the other hand, the analyses have been run for the global MA, and for the specific subscales, which bring the reader the possibility to examine its functioning either when the subscales are considered grouped together or isolated. In our opinion, taken together, the results found in our sample support the use of the SEMA as a useful instrument to measure MA in children throughout elementary school years, taking in mind the limitation already mentioned given by the fact that one of the subscales included specific math problems.

- line 160-161: Please report the words and/or pictures that made up the response scale.

We have provided the specific words used for each response option and informed that each option was illustrated with a cartoon face, that has been reproduced with the permission of the original authors in the SF1.

I know the faces they use in their picture scale and I find them to be difficult to interpret – **did you test that children understood the pictures well? Were both the words and pictures available to children when they responded or just the pictures?**

As introduced in (line 263, page 11), the SEMA was administered in an one-to-one interview and the children had the chance to ask if they did not understand any aspect concerning the items meaning or the scale. In addressing this issue, we have talked to the members of our staff who administered the questionnaire and they told us that children in general answered with no hesitation, giving the impression that they clearly understood the instructions given.

Results
- line 204: Can you please include a table that lists all of the items and their means, standard deviations, skewness, and kurtosis? And maybe means and standard deviations by grade level? I think including the original SEMA item as well as the Spanish translation would be most transparent for all readers.

Following the reviewer’ suggestion, we have introduced the required details in a new table (see Table 1, page 15).

- line 204-208: I’d like to know more about the items that you found to be problematic and what information you used to determine that they had low variability – was there a particular cut off? **Did these items correlate with other items on the scale?**

In (lines 270-284, page 12) we have clarified our decision of removing items 2, 4, 6, 12, and 13 based on the distribution of scores.

Regarding the correlation of each removed item, we have computed the item-test correlation and the Cronbach’s alpha with and without them for younger and older students:

| **Younger students** | | | |
| --- | --- | --- | --- |
| Item | Item-test correlation | Cronbach’s alpha of the final scale | Cronbach’s alpha of the scale with the item |
| 4 | .34 | .68 | .70 |
| 6 | .22 | .68 | .68 |
| 12 | .28 | .74 | .74 |
| **Older students** | | | |
| 2 | .43 | .70 | .72 |
| 4 | .40 | .70 | .72 |
| 6 | .24 | .70 | .70 |
| 12 | .44 | .78 | .79 |
| 13 | .37 | .78 | .79 |

- line 221 (and a few other places): You report p < .000, which is not possible, probably needs to be p < .001.

Sure, we have inserted this modification when necessary, throughout the text.

- line 222-223: You report the partial eta squared, which is useful, but for the gender difference can you report d as well so that is easier to compare with other studies? (same on line 238). You have a large sample size so I am guessing these ds are quite small.

Reviewer 2 pointed out that the *t*-tests on gender were not needed alongside reporting the main effect of gender from the ANOVAs; so, we have eliminated the *t*-test analyses (and consequently the possibility to report *d*). However, to help the readers to understand the meaning of partial eta squared we have introduced the following sentences: “The results indicated that grade and sex explain 2.5 and 0.7% of the SEMA scores, respectively” (lines 290-291, page 12); “The results indicated that grade and sex explain 5.7 and 0.7% of the numerical processing anxiety factor” (lines 299-300, page 13); and “The results revealed that gender explain 0.5% of the subscale scores” (lines 309-310, page 13).

- line 265 and 268: Can you include a range of values for the factor loadings? Were there any that were low (< .3, or .4)?

The values for the factor loading ranged from .47 to .63, and from .39 to .65, for Numerical processing Anxiety and Situational and Performance Anxiety scales respectively (lines 369-370, page 17).

- line 269: Was the 2-factor model a better fitting model than the one-factor model? I typically use the Satorra-Bentler statistic in MPlus, I am not sure if you can get this in R.

We thank the reviewer for the suggestion of using Satorra-Bentler statistic. The Package ‘SBSDiff’ (the RStudio package used to apply the Satorra-Bentler test) specifies that this test only runs using the estimator maximum likelihood (ML) and our estimator is "ULS" for unweighted least squares. Our decision to choose the ULS estimator is based on the paper published by Forero, Maydeu-Olivares, and Gallardo-Pujol (2009), where the authors found that ULS provides more accurate results for ordinal variables than the other estimators. Moreover, we have studied other statistics to compare fitting models in RStudio (such as BIC and AIC), but we found the same obstacle: they only work with ML. As a result, we used the fit indices (CFI, TLI, RMSEA, and SRMR) to decide which model (one- versus two- factor) show a better adjustment.

Attending to the fit indices brought out for the different models, both could be plausible. In the new version of the manuscript, we have paid more attention to the adequacy of broad versus narrow measures of MA in connection with math performance. This issue has been raised in the Introduction (lines 160-171, page 7), and the Discussion section (lines 505-518, pages 24-25, and lines 564-603, pages 27-28).

- Table 5: Because grade is a categorical variable with 4 categories, it is probably more appropriate to dummy code it in the regression analyses than to treat it as a continuous variable.

Following the reviewers’ suggestion, grade was treated as a categorical-ordinal variable.

- Table 5: How is gender coded in the models? I am asking because it is a statistically significant predictor and I don’t know if that means boys or girls had higher math scores.

Gender was coded as 0 for girls, and 1 for boys. This information has been provided in line 431, page 19.

- line 350: To test whether the relations are different by grade level, a regression analysis with an interaction term between grade and math anxiety would be able to statistically compare if the size of the relations across grades.

We thank the reviewer for the interesting suggestion. Following the recommendation, we have introduced the interaction term analyses for school stage and math anxiety (lines 460-485, pages 22-23), with significant effects between school stage and global MA to predict math grades, and also between school stage and the Situational and Performance factor to predict math grades. The new analyses indicate that higher scores on math anxiety are associated with lower math grades for younger and older students; but this association is stronger for younger than for older students.

Discussion
- line 377 paragraph: The grade issue here – though you discuss it, it is impossible to tease apart why you found these grade differences, and, if anything, the fact that you found them for the subscale that has math problems and not the other subscale lends some support to the explanation that **it is because the items have math problems, not because of any developmental differences between students in different grade levels**. In addition, this issue arises when interpreting the relations between math anxiety and performance for younger compared to older students. It is likely that what is being measured in younger students is slightly different from what is being measured in older students given the relative ease of the items for the older students. Thus, it does not surprise me that **the relation is stronger in younger students where you can really see how they feel in response to more difficult math. Again, this difference in relations may be due to the math problems used in the items, not to actual differences in the underlying construct of math anxiety**, and it makes it difficult to interpret this finding.

We thank the reviewer for this caution. As said before, we have highlighted this limitation in the new version of the manuscript.

It might help to run the analysis with the **two subscales separately and see if the relations are weaker for older children only for the numerical processing factor and not for the situational and performance factor**.

As has been mentioned, the suggested analyses were introduced in the results section (lines 459-486, pages 22-23), and discussed later at lines 604-621, page 28-29.

Regardless, I think that using this scale across multiple ages may be in appropriate given the content of the scale, and it might be best to remove any grade analyses from the paper given difficulty in interpreting these findings and analyses may need to just be run within grade where we can be more confident that the responses mean the same thing.

As it was said before, we have decided to keep the grade differences analyses because of its informative value, with caution about the interpretation of the results. Although we haven’t run all the analyses within grade, following your previous suggestion, we have divided the sample into younger (3er and 4^th^ grade), and older (5^th^ and 6^th^ grade) children to ascertain the predictive power of MA on math performance depending on the school stage. Introducing the MA*school stage interaction has brought out interesting results: The predictive power of MA on math performance only proved significant for both global MA and Situational and Performance factor, so that we found a weaker association between MA and math achievement in older children compared to younger ones. Importantly, the association of Numerical Processing anxiety with Fluency standardized test did not vary across school stage (please, see Results, lines 473-477, page 23). This latter result suggests that the Numerical Processing anxiety factor is useful to assess MA in early as well as upper elementary school, which extends the usefulness of the SEMA beyond the original age range.

- line 393 paragraph: Can you talk about **how the size of the gender difference you found compares to that found in other studies**? **And why the gender difference may have gone away when general anxiety was covaried out?**

- line 461: Also mention here that the gender differences were not apparent when general anxiety was covaried out.

In the new analyses run comparing average levels of MA for boys and girls, once we covaried trait anxiety, all the previous differences were gone. In the Discussion section (lines 534-546, pages 25-26), we have referred to the effect sizes of the differences as small, which disappeared when general anxiety was controlled, similarly to the study by Ganley and McGraw (2016), and explained this result based on the mediational study developed by Szczygiel (2020), in which it was proved a mediational pathway from gender to MA via general anxiety.
