# Supplementary material for: Assessing math anxiety in elementary schoolchildren through a Spanish version of the Scale for Early Mathematics Anxiety (SEMA)
Source: PLoS One. 2021 Aug 5;16(8):e0255777. doi: 10.1371/journal.pone.0255777 (PMC8341591; doi:10.1371/journal.pone.0255777)
Supplement: Attachment — Submitted filename: Rebuttal letter_1907.docx [file pone.0255777.s004.docx]

Dear editor,

We are very grateful for having given another opportunity to revise our manuscript entitled “Assessing math anxiety in elementary schoolchildren through a Spanish version of the Scale for Early Mathematics Anxiety (SEMA)” and to improve the quality of the paper. In this revised version, we have carefully considered the reviewers’ comments and suggestions. As instructed, we have replied to each comment raised by the two reviewers (the reviewer’s comments have been kept in black ink whereas our answers are in blue color), and also uploaded the revised manuscript in two versions: one marked-up copy highlighting the changes made to the original version and another unmarked version of the revised paper without tracked changes.

The reviewers’ comments were very specific and detailed, and we thank them for their time. After addressing all reviewers’ concerns, we feel of the paper has improved significantly and we hope it can reach the quality standards for publication in Plos One journal.

Best regards,

Dr. Noelia Sánchez-Pérez

**Reviewer #2**

In line with the most recent APA guidelines, presumably all in-text citations involving more than two authors should be changed to “first author et al.”?

We thank the reviewer for the reminder about the changes in the 7th edition of the APA Publication Manual. Following its guidelines, the in-text citations have been changed.

Lines 131-132 - “(2) Formulation of the rating scale so that it is 132 clearly understood by children.” needs to be reworded.

We reworded the sentence this way “use of a rating scale whose options have a clear meaning to children”.

Line 679 – “CLARK-CARTER” is unnecessarily capitalised.

The error was amended.

Line 357 – I would remove the word “usually”.

Removed.

**Reviewer #3**

- paragraph starting line 270: It would be useful to see the item wording for the items that were removed due to having low variability. Perhaps still include them in Table 1? I wondered if you could talk more conceptually about why they might have had such low rates of agreement of anxiety - were these especially easy math problems, for example? I’m still not sure I agree with removing them unless it was part of your goal to develop a scale with fewer items, which was not stated prior to this. They all seem to be correlated with the other items suggesting they do measure the same construct, but are just picking up on the kids with really high levels of math anxiety. (item 6 is perhaps borderline – it seems to have a pretty low item-total correlation at both age groups).

The removed items have been included in Table 1 (page 15-16) and marked with asterisks. We have referred to the content of the items as possible explanations for the floor effect found and have supported our decision of not including these items in further analyses on the work by Terwee et al., 2007, in which the absence of items with ceiling or floor effects is considered one of the quality criteria for the measurement properties of health status questionnaires (page 12, lines 280-292 from unmarked version of the revised paper without tracked changes).

- line 288: You say “analyses” – can you say if this was ANOVAs or something else and mention what was included in the model before going into the model results?

We have clarified the expression in the text by introducing “ANOVAs analyses” (page 13, line 297).

- line 295: I think you could include the d here for the gender difference (and in the places where there are gender differences reported). The d is not tied to the t-test and can be calculated on its own based on means and standard deviations.

We thank the reviewer for the suggestion. We have calculated the C*ohen's d* and introduced the results in the reported gender differences (page 13, line 304-305; 315-316; 322-323; 340-341).

- Table 1: Can you make it more clear which subscale each item belongs to? (even just a note at the bottom of the table would help)

We have specified the composition of two scales by introducing the sentence “Original items were obtained from Wu et al., (2012): items 1 to 10 constitute the Numerical Processing Anxiety factor; items 11 to 20 belong to the Situational and Performance Anxiety factor” at the bottom of the Table 1 (page 16)

- line 384: I’m not really sure what to take away from your CFA analyses – would you argue that a 1-factor or 2-factor model is what researchers should use? Or that both are reasonable to use? Is a correlation of .84 between 2 subscales of the same construct reasonable? How does this compare to the original SEMA?

We thank the reviewer for pointing to the need to further interpret the CFA analyses. In the Discussion section (page 25, lines 539-544), the meaning of the two factors found is referred to and have put in connection with previous literature. The issue concerning the strong correlation between the two factors is addressed in page 26 (lines 548-560). We based our interpretation on the work by Henschel and Roick (2020) in which the high correlation between the factors found in other instruments derived from the MARS is attributed to the fact that the MARS and derived questionnaires tap a unique dimension of math anxiety, that is, the affective component.

- paragraph starting line 406: I found this paragraph a bit confusing to follow because you hadn’t told me what was in each model before you told me results and you talked about the second model (with the covariates and math anxiety) before talking about the first model (with just the covariates), which was sort of backwards order to how the models were run. It also was not made clear that the F, p, and R2ajusted reported were for the whole model – it was written as though these were part of the results just for math anxiety.

We have reworded the paragraph to make clear the two regressions tests, the two steps and their results (page 19-20, lines 414-432)

- line 426: Why was grade a potential covariate tested for these models but not for the models predicting math grades? In my previous review I suggested treating grade as a categorical variable instead of a continuous variable, but it doesn’t look like you did that in the tables reported in this section as grade would need to be parsed into 3 pairwise comparisons using dummy coding.

We thank the reviewer for the question and reminder. We have tested the potential effect of grade in children’s math grades and the results brought out grade differences, with 3^rd^ graders obtaining higher scores than 4^th^ graders (page 19, line 406-409). Consequently, grade was introduced as a covariate in further analyses.

- line 446: Mention that numerical processing anxiety did not predict calculation skills.

It was clarified in the text (page 22, lines 467-468).

- line 448: It is unclear if your results in the regression analyses for the 2 factors are different from the zero-order correlations because they are highly correlated with one another or because the covariates are included in the model. Can you add a model where just the 2 math anxiety factors are predictors without the covariates? This would help to tease apart whether it’s including the subscales together or if its’ including the covariates that leads some of them to not be significant despite significant zero-order correlations for both.

We have run regression analyses to predict calculation and fluency abilities, and introduced the paragraph with the following results: “To determine whether the weaker relationship between the situational and performance anxiety factors and the standard tests that were observed in the regression models compared with the correlation analyses, were caused by either the correlation between the two factors or the inclusion of the covariates in the model, we performed an additional regression analysis. As shown in Table 7, the numerical processing anxiety factor did yield a significant contribution to Calculation and Math fluency abilities, but the situational and performance anxiety factor did not” (page 22, lines 462; Table 7, page 23)

| DV: Calculation scale | F | R^2^_adj_ | Standardized β | *p* |
| --- | --- | --- | --- | --- |
| Model | 26.41*** | .05 |  |  |
| Numerical processing anxiety factor |  |  | -.22 | < .001 |
| Situational and performance anxiety factor |  |  | -.02 | .706 |
| DV: Fluency scale | F | R^2^_adj_ | Standardized β | *p* |
| Model | 54.29*** | .11 |  |  |
| Numerical processing anxiety factor |  |  | -.32 | < .001 |
| Situational and performance anxiety factor |  |  | -.01 | .786 |

Did the interaction analyses include the same covariates as were previously included?

Thanks to the author question we realized that we included gender as a covariate for math grades when in fact they were not significantly related. Consequently, we run again the analyses for math grades. The interaction results did not change, as it is shown in page 23, lines 492-517).

- line 519: Can you say more about what you think your CFA results mean for the construct of math anxiety? Are these separate components meaningfully different?

As expressed above, this issue has been addressed in the Discussion section, pages 26 and 27.

- line 538: You mention that the gender difference in global MA and numerical processing anxiety went away, but didn’t it go away for situational/performance anxiety as well?

We thank the reviewer for pointing out our mistake: we have now specified that the gender differences disappeared for global MA and both factors with the sentence “when the general levels of anxiety were controlled for, the differences found for global MA, the numerical processing, and situational and performance anxiety factors disappeared” (page 27, lines 576-578).

- line 584: The way this sentence is written implies that numerical processing anxiety predicted both of the standardized math test outcomes, but it only predicted **one of them.**

As the reviewer has noted, numerical processing anxiety predicted fluency scores, but not calculation skills. We have clarified the specific scale in the discussion section (page 29, lines 622-624).
